# Use of apple pomace, glycerine, and potato wastewater for the production of propionic acid and vitamin B12

**DOI:** 10.1007/s00253-022-12076-w

**Published:** 2022-07-26

**Authors:** Kamil Piwowarek, Edyta Lipińska, Elżbieta Hać-Szymańczuk, Vitaliy Kolotylo, Marek Kieliszek

**Affiliations:** Department of Food Biotechnology and Microbiology, Institute of Food Sciences, Warsaw University of Life Sciences — SGGW, Nowoursynowska 159C, 02-776 Warsaw, Poland

**Keywords:** Apple pomace, Glycerine, Potato wastewater, Side-streams, Residues, Utilization, Propionic acid, Vitamin B12

## Abstract

**Abstract:**

Propionic acid bacteria (PAB) are a source of valuable metabolites, including propionic acid and vitamin B12. Propionic acid, a food preservative, is synthesized from petroleum refining by-products, giving rise to ecological concerns. Due to changing food trends, the demand for vitamin B12 has been expected to increase in the future. Therefore, it is necessary to look for new, alternative methods of obtaining these compounds. This study was conducted with an aim of optimizing the production of PAB metabolites using only residues (apple pomace, waste glycerine, and potato wastewater), without any enzymatic or chemical pretreatment and enrichment. Media consisting of one, two, or three industrial side-streams were used for the production of PAB metabolites. The highest production of propionic acid was observed in the medium containing all three residues (8.15 g/L, yield: 0.48 g/g). In the same medium, the highest production of acetic acid was found — 2.31 g/L (0.13 g/g). The presence of waste glycerine in the media had a positive effect on the efficiency of propionic acid production and P/A ratio. The concentration of vitamin B12 obtained in the wet biomass of *Propionibacterium freudenreichii* DSM 20271 ranged from 90 to 290 µg/100 g. The highest production of cobalamin was achieved in potato wastewater and apple pomace, which may be a source of the precursors of vitamin B12 — cobalt and riboflavin. The results obtained show both propionic acid and vitamin B12 can be produced in a more sustainable manner through the fermentation of residues which are often not properly managed.

**Key points:**

• *The tested strain has been showed metabolic activity in the analyzed industrial side-streams (apple pomace, waste glycerine, potato wastewater).*

• *All the side-streams were relevant for the production of propinic acid.*

• *The addition of waste glycerine increases the propionic acid production efficiency and P/A ratio.*

• *B12 was produced the most in the media containing potato wastewater and apple pomace as dominant ingredients.*

## Introduction

Propionic acid bacteria (PAB) are Gram-positive, nonsporulating rods. They are classified as anaerobes or relative anaerobes, and their cells exist singly, in pairs, or in chains. In the presence of oxygen, PAB exhibit pleomorphism (multiformity) taking up various shapes (e.g., club-shaped, Y-shaped, or V-shaped), while in anaerobic conditions, they appear as small, short sticks, resembling cocci in microscopic images (Piwowarek et al. 2018). The optimal temperature for the growth of PAB is 30–37 °C, and the optimal pH is 6.0–7.0. PAB are characterized by high nutritional requirements. Apart from carbon (glucose, glycerol, or lactic acid) and nitrogen (ammonium salts, amino acids, peptides), they need appropriate microelements (e.g., iron, manganese, magnesium, cobalt, copper) and vitamins (B2 — riboflavin, B3 — nicotinamide, B5 — pantothenic acid, biotin — B7). *Propionibacterium freudenreichii* is a source of industrially valuable metabolites including propionic acid and vitamin B12. What is more, this species has GRAS status — both metabolites of these bacteria and living cells can be used in the food and feed industry.

Propionic acid is primarily used in the food and feed industry as a preservative — it can inhibit the growth of yeasts and molds. In addition, this compound and its derivatives act as substrates in the production of cellulose fibers, herbicides, perfumes, and pharmaceuticals (paracetamol). At present, propionic acid is produced in industries mainly using two petrochemical processes — Reppe and Larson (Gonzalez-Garcia et al. 2017; Eş et al. 2017). The market dominance of synthetic propionic acid can be attributed to its production costs. Propionic acid obtained via chemical synthesis is almost two times cheaper than bio-based propionic acid (Baumann and Westermann 2016). Microbial production of propionic acid is mostly limited by the lower production efficiency, the need to purify propionic acid from postculture fluid, and the costs of fermentation medium, which account for more than half of the production costs (Yang et al. 2018). The use of PAB would allow propionic acid synthesis independent of petroleum production, which would have tangible benefits due to constant fluctuations in the price of crude oil and declining raw material resources.

Vitamin B12 is unique because it is synthesized only by bacteria, e.g., *Propionibacterium freudenreichii* (Martens et al. 2002). In the human diet, animal-derived products are the main source of cobalamin. Vitamin B12 is responsible for the synthesis of blood cells in the hematopoietic system, and also participates in DNA and RNA synthesis in erythroblasts. In addition, this vitamin is involved in the metabolism of fats and carbohydrates, transformation of purines and pyrimidines (DNA metabolism), and conversion of folic acid into tetrahydrofolate, thereby contributing to maintaining the stability of the human genome. Furthermore, vitamin B12 is essential for the proper functioning of the nervous system as it takes part in the formation of myelin sheaths and nerve transmitters. Due to these vital functions, the deficiency of vitamin B12 results in cognitive, neurological, and hematological disorders (Kolling et al. 2004; Luggen 2006; Smith et al. 2007; Green et al. 2017). According to recent reports (Green et al. 2017), vitamin B12 deficiency, which was previously thought to be very rare except in strict vegans, is common even among vegetarians (e.g., lactovegetarians), and is therefore becoming a global problem. Considering the growing environmental and animal welfare concerns, and thus changing food trends (increasing popularity of vegetarian and vegan diets), it can be expected that the demand for vitamin B12 supplements and vitamin B12-enriched plant products will increase in the coming decades. A significant shift in consumption patterns from animal protein to plant-based protein may occur in the future. This will be a huge challenge for food producers because, regardless of the source, the nutritional value of food products must be maintained, and the most important nutrient that cannot be obtained from any plant product is vitamin B12 (Green et al. 2017).

A possible solution to overcome the production costs of PAB metabolites is the development of a cheap medium, such as a medium entirely made from industrial residues, which would guarantee efficient metabolism of propionic acid bacteria (Vidra and Németh 2018). Huge amounts of residues generated consist of biogenic elements and organic compounds, which makes their disposal expensive and troublesome. As a result, a large proportion of the residues ends in landfills without any specific use. Some by-products require new solutions for their valorization. Fermentation seems to be a cheap, energy-saving, and sustainable alternative for managing residues. Apple pomace, potato wastewater, and waste glycerine are considered as materials affecting the environment. Nevertheless, they have a rich composition, so it is worth considering them as raw materials for further processing.

Juice production (fruit pressing) results in up to 5.5 MMT of residues, mainly pomace. However, only 20% of pomace is recovered for further use, and the rest is landfilled, composted, or incinerated. It is assumed that several million tons of pomace are produced annually worldwide. For example, in the USA, about 1 million tons of pomace is generated annually, while Brazil and Germany account for 800 thousand and 250 thousand tons, respectively. In Poland, the production of apple pomace is in the range of 400–600 thousand tons. Fruit pomace is unstable due to the high content of water and biologically active ingredients (e.g., sugars, elements, and vitamins). A certain portion of fruit residues is preserved in the place of its generation, for example, by drying. Dried pomace is used as an ingredient of animal feed (Shalini and Gupta 2010; Lyu et al. 2020). Nevertheless, not all processing plants can afford the process of fruit residues preservation owing to high investment (for equipment) and operating costs (energy, water). A good solution for managing fresh (unpreserved) residues is to use it by employing microorganisms. Another type of industrial side-stream with negative environmental impact is potato wastewater — generated during the production of potato starch. It is estimated that the processing of 1000 tons of potatoes results in about 600 tons of potato wastewater. Europe accounts for approximately 1.7 million tons of potato starch every year. Potato wastewater has no specific applications. It is often diluted with water and then sprinkled on fields (source of nitrogen) as a fertilizer. However, due to its high pollution load (high chemical and biological oxygen demand) (Kieliszek et al. 2020; Kot et al. 2020), the application of this residue as a fertilizer is not favorable for its disposal, and may even lead to the eutrophication of water and deterioration of soil fertility. Industrial residues also include waste glycerine — by-product generated from the production of biodiesel, a fuel that can be used in conventional engines. The application of glycerine is limited by the high production costs, which are largely due to the necessity to utilize the resulting residues. A large part of glycerine is glycerol, and the remaining part consists of impurities such as methanol, soaps, and traces of heavy metals (Kot et al. 2017). Prior to its industrial application, glycerine must undergo a purification process consisting of deodorization, bleaching, and ion exchange — to obtain pure glycerol for use in pharmaceutical, cosmetic, food, and chemical industries. As this process is expensive and cannot be afforded by small industrial plants (Quispe et al. 2013), the possible solution for managing the glycerine fraction is to use it as a component in culture media for microorganisms.

The aim of this study was to optimize the production of propionic acid and vitamin B12 by *P. freudenreichii* DSM 20271 using media containing different proportions of industrial residues — apple pomace, glycerine, and potato wastewater. It was also investigated whether growth conditions favoring simultaneous efficient production of acid and vitamin B12 by PAB can be achieved. The obtained results showed which residues were found to be better for the synthesis of particular metabolites.

## Materials and methods

### Biological material

The *P. freudenreichii* DSM 20271 strain used in the experiment was obtained from the German Collection of Microorganisms and Cell Cultures GmbH, Leibniz Institute. The bacteria were stored in VL (BTL, Poland) medium at 4 °C and frozen in a glycerol environment (− 80 °C).

Apple pomace was obtained from Hortex sp. z o.o. (Poland, Warszawa), and waste glycerine from Orlen S.A. (Poland, Płock). Potato wastewater was prepared from potatoes of the Irga variety in laboratory conditions according to the methodology developed on the basis of the course of individual stages of potato starch production (Kot et al. 2017). Apple pomace (derived from various Polish varieties of apples) was frozen (− 20 °C), glycerine was stored at 6–8 °C, and potato wastewater was stored in glass bottles at room temperature until use.

The concentration of sugars in apple pomace and potato wastewater was determined by the enzymatic method using the Sucrose/D-Fructose/D-Glucose Assay Kit obtained from Megazyme (Bray, County Wicklow, Ireland), according to the manufacturer’s instruction. The total nitrogen content in residues was determined by applying the Kjeldahl method. The content of glycerol in glycerine was 80%.

### Growth media

The VL medium used for propagating the bacteria consisted of glucose (1 g/L), meat extract (3 g/L), peptone (10 g/L), sodium chloride (5 g/L), yeast extract (5 g/L), and L-cysteine hydrochloride (0.40 g/L). The medium was sterilized in an autoclave (117 °C, 20 min) before inoculation. The pH of the medium was set to 6.5–7.0 using NaOH.

The composition of the media used for the experiment is presented in Table 1. An appropriate volume of water and/or potato wastewater (depending on the medium variant) was added to an appropriate amount of apple pomace in glass bottles. The whole solution was heated (extracted) for 25 min at 75 °C under stirring. After heating, insoluble materials were removed from the solution by filtration. The extract was centrifuged to remove residual solids (3500 rpm, 10 min), and the supernatant was used to prepare the experimental media. For variants with glycerine, glycerine was weighed out as mentioned in Table 1. The media were sterilized at 121 °C for 20 min, and the pH was adjusted to 7.0 using NaOH. L-cysteine hydrochloride (0.40 g/L) was added to the media to create anaerobic conditions.

**Table 1:** Composition of side-streams in the culture media

Medium	Apple pomace		Potato wastewater		Waste glycerine	
	%	g/100 mL	%	mL/100 mL	%	g/100 mL
1	100	100	0	0*	0	0
2	0	0	100	100	0	0
3	0	0	0	0*	100	2.5
4	50	50	50	50*	0	0
5	50	50	0	0*	50	1.25
6	0	0	50	50*	50	1.25
7	33.3	33.3	33.3	33.3*	33.7	0.8425
8	66.7	66.7	16.7	16.7*	16.7	0.4175
9	16.7	16.7	66.7	66.7*	16.7	0.4175
10	16.7	16.7	16.7	16.7*	66.7	1.6675

^*^Distilled water was added to a volume of 100 mL

The concentrations of sugars and nitrogen in the experimental media were determined as described above. The content of glycerol was determined by the enzymatic method using the Glycerol Assay Kit (Megazyme), according to the manufacturer’s instruction.

### Culture conditions

Inoculation was carried out in Erlenmeyer flasks, containing 100 mL of VL liquid medium for 24 h, under static conditions at 30 °C. The resulting inoculum was used to inoculate liquid experimental media in a given series in the following way. The inoculation medium (25 mL) was collected in 50 mL tubes, and the inoculum cultures were centrifuged (10,000 rpm, 10 min). After centrifugation, the supernatant was decanted from the pellet, and the obtained biomass was suspended in a suitable experimental medium (25 mL), then it was poured into an appropriate experimental medium (225 mL). The inoculum accounted for 10% of the volume of the production medium.

The experimental cultures were carried out in Erlenmeyer flasks containing 250 mL of the liquid medium under static conditions. Cultures were maintained for 120 h at 30 °C. The media were neutralized with 10% NaOH every 24 h to optimize the pH for PAB, if necessary. Samples for analysis were taken at 0–120 h time intervals, every 24 h. For each variant of the medium, the cultures were carried out simultaneously in three flasks (flask 1 — 24 and 48 h, flask 2 — 72 and 96 h, flask 3 — 120 h).

### Analytical methods

The yield of cellular biomass was determined by weighing. The content of sugars, glycerol, and nitrogen in the experimental media was determined as described above.

The analysis of propionic and acetic acids was carried out using a gas chromatograph equipped with a flame ionization detector (Trace 1300 Gas Chromatography, Thermo Scientific, Waltham, MA, USA). Free organic acids were released from propionic and acetic acid salts (resulting from regular alkalization of the media during fermentation) by adding 25% sulphuric acid (VI) (Chempur, Poland) to postculture fluids. The fraction of carboxylic acids was extracted from the media using a mixture of hexane (Chempur, Poland) and diethyl ether (Chempur, Poland) (1/1, v/v). Chromatographic separation was carried out on ZB-WAXplus column (30 m × 0.25 mm × 0.25 µm). Qualitative analysis of acids was performed by comparing the retention times of the tested samples with standards. Quantitative analyses were carried out using the analytical standard (undecanoic acid — Sigma-Aldrich, USA). The yield of acids was calculated by dividing the mass of propionic or acetic acid obtained by the respective mass of carbon sources consumed and was expressed in g/g. Volumetric productivity was calculated by dividing the concentration of propionic acid or acetic acid obtained at the end of fermentation by the time elapsed to reach that concentration and was expressed in g/L/h.

Quantitative determination of intracellular vitamin B12 produced by *P. freudenreichii* was carried out using a microbial assay in a microplate format, known as VitaFast®Vitamin B12 assay (R-Biopharm, Darmstadt, Germany). The content of vitamin B12 in cellular biomass was determined at 120 h of culture for each variant. To extract vitamin B12 from biomass, 20 mL of acetate buffer (Sigma-Aldrich, USA), 250 µL of 1% NaCN (Sigma-Aldrich, USA), and 300 mg Taka-diastase enzyme (Sigma-Aldrich, USA) were added to the wet biomass of the tested strain, respectively. The mixture was incubated in the dark at 37 °C for 1 h, and the samples were shaken every 10 min. After incubation, distilled water was added to the samples to a final volume of 40 mL. Vitamin B12 was extracted in the water bath in the dark (95 °C) for 30 min. The samples were shaken continuously during extraction. After extraction, the samples were cooled to a temperature below 30 °C and centrifuged (10,000 rpm, 4 °C, 10 min). The clear supernatant was diluted with sterile redistilled water (1:1000 or 1:10,000). The obtained extracts were filtered through sterile syringe filters. Then, 150 µL of the medium (attached to the test) and 150 µL of the vitamin B12 standard (attached to the test, standard curve — 0.00, 0.030, 0.060, 0.090, 0.120, and 0.180 µg/100 g) or 150 µL of the extract were added to the wells (coated with *Lactobacillus delbrueckii* subsp. *lactis*) on the plates. The prepared plates were incubated for 48 h in the dark (37 °C). The biomass yield of *L. delbrueckii* was dependent on the amount of vitamin B12 in the culture environment. The bacteria continued to grow until the entire vitamin B12 in the sample was consumed — the more vitamin in the tested sample, the greater the growth of *Lactobacillus* bacteria. Correlation of bacterial growth with the concentration of extracted vitamin B12 was expressed as turbidity (measured by spectrophotometry using a microplate reader and a 540-nm filter) (ELISA Plate Reader, Thermo Fisher Scientific, USA). The content of vitamin B12 in the cellular biomass of PAB was determined from the standard curve. The samples for analysis were performed in a sterile manner (using laminar chamber, syringe filters, sterile tubes, etc.). The content of vitamin B12 in bacterial biomass was calculated using the formula:

$$B12 \left[\frac{\upmu g}{100g}\right]= \frac{\mathrm{Conc}.\mathrm{ standard curve }\cdot \mathrm{ Dilution factor }}{\mathrm{Sample weight}}$$

The vitamin B12 content in the inoculum was measured — 37 µg/100 g wet biomass, this value was subtracted from the concentrations measured in the tested samples.

### Statistical analysis

All experiments were performed in triplicate. Mathematical and statistical calculations were done in Excel 2013 (Microsoft) and STATISTICA 10.0 (StatSoft. Inc.) software, respectively. Due to the need for multiple combinations of the three selected substrates, the mixture design method of DoE (Design of Experiment) was used as a tool to optimize the composition of residues in the culture medium. Simplex plans were used for ternary mixtures. The significance of differences between the mean values was analyzed by one-way analysis of variance and Tukey’s test. The level of significance was set at 0.05.

## Results

### Characteristics of industrial residues, consumption of carbon sources, bacterial growth

Potato wastewater (M2 — medium 2) was characterized by a higher nitrogen content compared to apple pomace (M1) (Table 2). A L of potato wastewater had 2.91 g of nitrogen, while in the pomace, nitrogen was present in the amount of 0.32 g/L. The total sugar content in the apple pomace was 23.15 g/L. The share of fructose was highest in the total sugar content (60%), glucose accounted for 40% (there was no sucrose). On the other hand, only 5.06 g of sugars was found in L of potato wastewater (Table 2). Glycerine contained neither sugar nor nitrogen. Glycerol content in waste glycerine fluctuated around 80% — in accordance with the manufacturer’s declaration.

**Table 2:** Comparison of glucose and fructose content (0 h and 120 h), nitrogen sources (0 h), and C/N molar ratios in the culture media

Medium	1	2	3	4	5	6	7	8	9	10
Glucose 0 h [g/L]	9.24 ± 1.17	2.40 ± 0.18	0.00 ± 0.00	5.09 ± 0.24	5.48 ± 0.22	1.32 ± 0.35	2.78 ± 1.69	6.45 ± 0.53	2.79 ± 0.60	2.71 ± 0.29
Fructose 0 h [g/L]	13.91 ± 0.28	2.66 ± 0.04	0.00 ± 0.00	7.33 ± 0.54	9.71 ± 0.35	1.57 ± 0.52	6.43 ± 0.40	10.94 ± 0.25	4.72 ± 0.38	4.20 ± 0.09
Total sugars (glucose + fructose) 0 h [g/L]	23.15 ± 0.89	5.06 ± 0.14	0.00 ± 0.00	12.42 ± 0.79	15.19 ± 0.57	2.90 ± 0.17	9.21 ± 1.49	17.40 ± 0.78	7.51 ± 0.23	6.91 ± 0.30
Glycerol 0 h [g/L]	0.00 ± 0.00	0.00 ± 0.00	25.77 ± 0.83	0.00 ± 0.00	13.13 ± 0.90	14.95 ± 2.50	9.92 ± 2.18	5.30 ± 0.65	5.10 ± 0.67	18.33 ± 2.00
Nitrogen 0 h [g/L]	0.32 ± 0.03	2.91 ± 0.10	0.00 ± 0.00	1.57 ± 0.06	0.15 ± 0.00	1.55 ± 0.01	1.08 ± 0.01	0.68 ± 0.00	1.98 ± 0.01	0.68 ± 0.01
C/N molar ratio	14.22:1	0.34:1	–	1.53:1	54.04:1	4.12:1	5.23:1	7.97:1	1.74:1	12.40:1
Glucose 120 h [g/L]	1.17 ± 0.11	0.08 ± 0.00	0.00 ± 0.00	0.02 ± 0.01	1.61 ± 0.53	0.02 ± 0.00	0.06 ± 0.01	0.00 ± 0.00	0.09 ± 0.01	0.05 ± 0.05
Fructose 120 h [g/L]	7.57 ± 0.15	0.30 ± 0.06	0.00 ± 0.00	2.18 ± 0.03	5.96 ± 0.40	0.81 ± 0.02	1.34 ± 0.12	0.98 ± 0.02	1.04 ± 0.13	3.31 ± 0.23

*C/N*, carbon/nitrogen

**Fig. 1:**
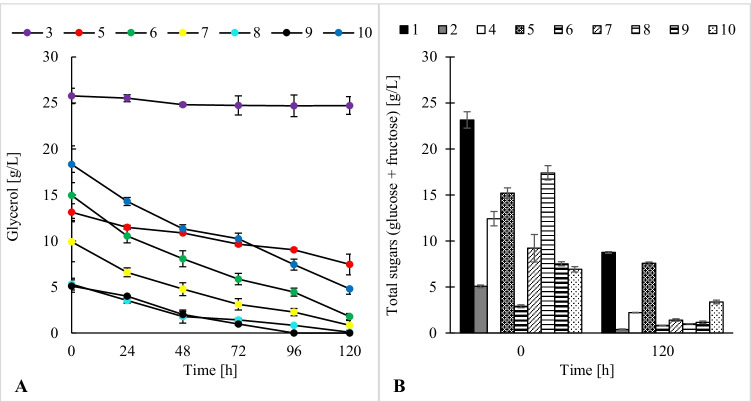
Consumption of glycerol (**A**) and sugars in total (**B**) by *P. freudenreichii* DSM 20271 strain in the culture media

The highest total concentration of sugars, mainly from apple pomace, was recorded in M1 (23.15 g/L), M4 (12.42 g/L), M5 (15.19 g/L), and M8 (17.40 g/L), while the starting content of carbohydrate in the remaining media variants was below 10 g/L (Fig. 1B, Table 2). The smallest amount of sugars was found in the medium containing only potato wastewater (M2, 5.06 g/L). The highest consumption (over 90%) was recorded in M2 (92.42%, 4.68 g/L) and M8 (94.34%, 16.41 g/L), while the lowest sugar update was observed in M5 (50%, 7.62 g/L) and M10 (51%, 3.55 g/L). Each tested medium, except for M3 (waste glycerine), contained two sugars — glucose and fructose. In most of the variants, fructose was the predominant sugar, and was almost 1.5- to twofold higher in concentration than glucose. The exception was M2 and M6, in which the ratio of glucose and fructose was 1:1. Depending on the growth environment, the amount of glucose consumed was in the range of 70–100%, whereas fructose — 21–91%.

In some media, along with apple pomace and/or potato wastewater, glycerine was also used, which acted as a source of glycerol for the synthesis of propionic acid by PAB (Fig. 1A, Table 2). The highest concentration of glycerol was found in M3 (25.76 g/L), M5 (13.13 g/L), M6 (14.95 g/L), and M10 (18.33 g/L), while in the remaining variants, glycerol was present at an amount of less than 10 g/L or completely absent (M1 and M2). In six of the variants containing waste glycerine, glycerol was consumed by the tested strain, while in M3 which contained only glycerine as the growth medium, glycerol was not utilized. The maximum utilization of glycerol, in most variants (M5, M6, M7, M8, M9, and M10), was noted at 120 h of cultivation (from 43 to 99%). The only variant where complete utilization of glycerol was recorded (at 96 h) was M9 (5.10 g/L). Glycerol were consuming by bacteria from the beginning of the cultivation, regardless of the medium variant (except for M3).

In M1, M3, M5, M8, and M10, the initial total content of carbon sources (sugars + glycerol) was above 20 g/L (23.15, 27.77, 28.32, 22.70, and 25.25 g/L, respectively). In M7, the initial content of sugars and glycerol was at a level of 19.13 g/L. In the remaining variants, the concentration of carbon sources was in the range of 5.06–17.84 g/L. These variations in the content of carbon sources between the media are due to the use of different proportions of industrial residues. The available carbon sources were not fully utilized in any of the tested medium variants. The highest percentage of utilization of total carbon sources (sugars + glycerol) by the tested strain was recorded in M2, M8, and M9 (92% (4.68 g/L), 95% (21.64 g/L), and 91% (11.47 g/L), respectively), while the lowest utilization (%) was recorded in M3 and M5 (58% (16.16 g/L) and 47% (13.30 g/L), respectively). The highest amount of carbon sources was used in M7 (16.91 g/L), M8 (21.64 g/L), and M10 (17.10 g/L), while the lowest in M2 (4.68 g/L), M4 (10.22 g/L), and M9 (11.47 g/L).

The variant containing the highest concentration of nitrogen was M2 (potato wastewater, 2.8 g/L), while trace amounts of nitrogen were found in M1 and M5 which were variants composed only of apple pomace and of pomace and glycerine, without the addition of potato wastewater (Table 2). The only variant which lacked nitrogen was M3. The different values of the C/N molar ratio in the analyzed media were related to the different proportions of residues, and thus different concentrations of sugars, glycerol, and nitrogen.

**Fig. 2:**
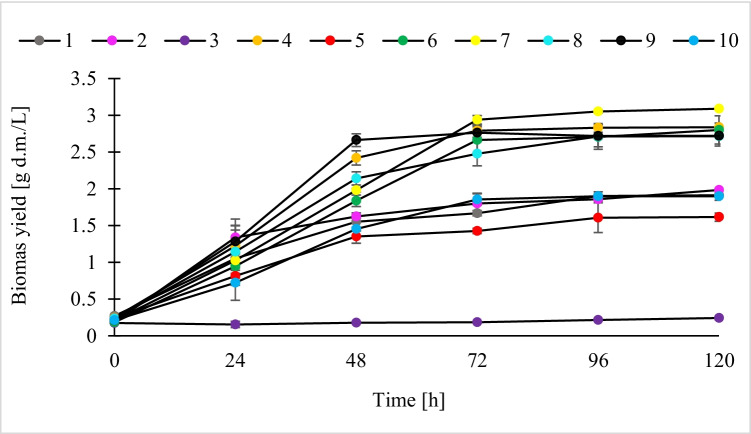
Growth of *P. freudenreichii* DSM 20271 strain in the culture media

The *P. freudenreichii* DSM 20271 strain grew in most of the tested media, except for M3 which contained only glycerine (Fig. 2). The highest biomass yield of the tested strain was obtained in M7 (3.09 g d.m./L), while the lowest in M5 (1.61 g d.m./L). Depending on the medium variant, the logarithmic growth phase lasted up to 48 (M5 and M9), 72 (M2, M4, M6, M7, and M10), or 96 h of culture (M1 and M8). Generally, the highest growth of *P. freudenreichii* DSM 20271 strain was found in variants containing potato wastewater (M4, M6, M7, M8, and M9), in which a biomass yield of over 2.72 g d.m./L was achieved. The exception was M10, which also contained potato wastewater, but the biomass yield obtained in this variant (1.90 g d.m./L) significantly differed from the other variants containing this material. In the remaining media exhibiting poor growth of the tested strain, the maximum biomass yield did not exceed 2 g d.m./L (M1 — 1.91 g d.m./L, M2 — 1.98 g d.m./L, M5 — 1.61 g d.m./L).

### Production of acids and vitamin B12

**Fig. 3:**
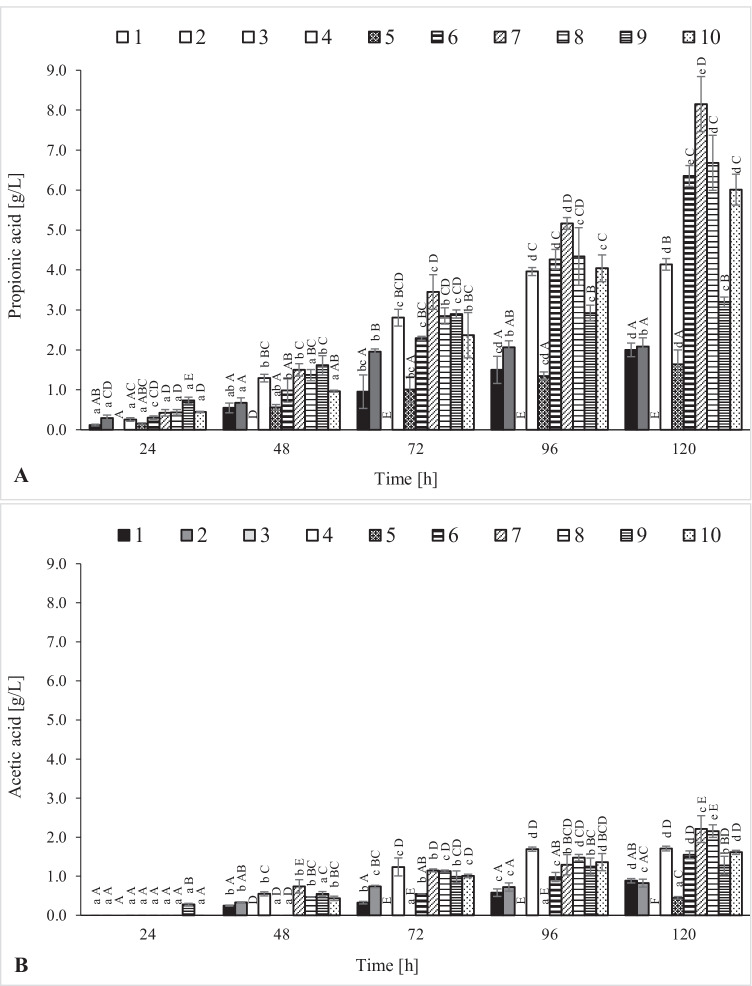
Production of propionic acid (**A**) and acetic acid (**B**) by *P. freudenreichii* DSM 20271 strain in the culture media. A, B, C, D, E — homogeneous groups of the influence of the type of medium on the production of propionic acid/acetic acid, the Tukey’s test (one-way analysis of variance); a, b, c, d, e — homogeneous groups of the influence of the cultivation time on the production of propionic acid/acetic acid, the Tukey’s test (one-way analysis of variance)

PAB produce propionic acid and acetic acid, which are the main metabolites of these bacteria and a by-product of fermentation. The *P. freudenreichii* DSM 20271 strain synthesized both these acids in most of the analyzed variants. The exception was M3 in which none of the acids was found, regardless of the time of the analysis. In all the media variants, propionic acid synthesis took place from the beginning of the cultivation (Fig. 3A). In M6, M7, M8, and M10, statistically significant production of propionic acid occurred up to 120 h of fermentation, while in other variants, the synthesis occurred up to 72 (M2 and M9) or 96 h (M4) of culture. The highest amount of propionic acid was found in M7 (8.15 g/L). Among the analyzed variants, M6 (6.36 g/L), M8 (6.68 g/L), and M10 (6.01 g/L) were also characterized by high production of propionic acid. On the other hand, the lowest concentration of propionic acid was found in M1, M2, and M5 (2.00, 2.09, and 1.64 g/L, respectively).

In most of the analyzed variants, the maximum acetic acid synthesis was recorded at 120 h of fermentation (Fig. 3B). In M2 and M9, the production of this metabolite lasted up to 72 h of cultivation (in M4 and M10 — up to 96 h). In variants 7 and 8, the highest acetic acid production was observed at 120 h of fermentation (2.21 and 2.16 g/L, respectively). In turn, the smallest amount of production was observed in M5 (0.45 g/L). The only variant in which acetic acid synthesis took place from the start of the fermentation was M9. In M5, acetic acid was synthesized only at 120 h of culture. In the remaining variants, acetic acid was identified from 48 h (M1, M2, M4, M7, M8, and M10) or 72 h (M6) of culture.

The highest efficiency of propionic acid production was achieved in M7 (0.48 g/g), which contained apple pomace, waste glycerine, and potato wastewater (Table 3). The highest productivity of propionic acid was also found in M7 (0.068 g/L/h). The lowest yield of propionic acid fermentation was recorded in M1 containing only apple pomace and M5 containing apple pomace and glycerine (0.14 and 0.12 g/g, respectively). The most efficient synthesis of acetic acid was observed in M2 containing only potato wastewater and M4 containing apple pomace and potato wastewater (0.18 and 0.17 g/g, respectively). In four out of 10 analyzed variants (M1, M2, M4, and M9), the weight ratio of propionic acid to acetic acid was approximately 2:1. The highest P/A ratio was recorded in M6 (4.11:1), and high P/A ratio was also found in M5 (3.69:1), M7 (3.68:1), and M10 (3.73:1).

**Table 3:** Comparison of fermentation parameters in the culture media and vitamin B12 production by *P. freudenreichii* DSM 20271. A, B, C, D, E, F — homogeneous groups of the influence of the type of medium on the production of vitamin B12, the Tukey’s test (one-way analysis of variance)

Medium	PA yield [g/g]	PA productivity [g/L/h]	AA yield [g/g]	AA productivity [g/L/h]	P/A ratio [g/g]	Vitamin B12 [µg/100 g wet biomass]
1	0.14	0.017	0.06	0.007	2.26:1	126.58 ± 2.93^A^
2	0.45	0.017	0.18	0.007	2.54:1	159.94 ± 12.29^B^
3	0.00	0.000	0.00	0.000	–	–^D^
4	0.41	0.034	0.17	0.014	2.42:1	289.80 ± 9.68^E^
5	0.12	0.014	0.03	0.004	3.69:1	90.80 ± 5.80^C^
6	0.42	0.053	0.11	0.013	4.11:1	160.65 ± 13.63^B^
7	0.48	0.068	0.13	0.018	3.68:1	100.50 ± 4.03^C^
8	0.31	0.056	0.10	0.018	3.10:1	121.94 ± 6.73^A^
9	0.30	0.027	0.11	0.011	2.51:1	242.31 ± 2.17^F^
10	0.35	0.050	0.09	0.013	3.73:1	138.33 ± 11.58^AB^

*PA*, propionic acid; *AA*, acetic acid; *P/A*, propionic acid/acetic acid weight ratio

In addition to acids, the content of vitamin B12 was determined in the wet biomass of *P. freudenreichii* DSM 20271 strain after 120 h of cultivation (Table 3). Interestingly, this compound was found to be produced in almost every variant, with the exception of M3, in which the tested strain did not show any metabolic activity. The highest cobalamin synthesis was found in M4 (289.80 µg/100 g wet biomass) which contained apple pomace and potato wastewater. M9 (242.31 µg/100 g wet biomass), the main ingredient of which was potato wastewater, was also characterized by efficient production of vitamin B12. The lowest amount of vitamin B12 was found in bacterial cell biomass obtained from M1, M5, M7, and M8 (126.58, 90.80, 100.50, and 121.94 µg/100 g wet biomass, respectively). All these four media contained very less or no potato wastewater.

## Discussion

### Characteristics of industrial residues, consumption of carbon sources, bacterial growth

The chemical composition of residues depends on the variety of the raw material, climatic and atmospheric conditions during cultivation, harvest time, and method used for raw material storage and processing. Magyar et al. (2016) observed that most of the sugars available in the apple pomace are glucose and fructose. In the apple pomace used by Piwowarek et al. (2021a), the total sugar content was 45.40 g/L. The share of fructose was highest in the total sugar content (53%), followed by glucose (42%) and sucrose (5%). A common feature of all apple pomace is the low nitrogen content (Magyar et al. 2016; Piwowarek et al. 2021a). In the potato wastewater used by Piwowarek et al. (2021a), the concentration of nitrogen was 2.14 g/L and sugars — 5.10 g/L. Similar values were found for the residues used in this study: apple pomace (total sugar content: 23.15 g/L, nitrogen content: 0.32 g/L), potato wastewater (total sugar content: 5.06 g/L, nitrogen content: 2.91 g/L). In this study, the molar ratio of carbon to nitrogen was 0.34:1 in potato wastewater and 14.22:1 in apple pomace. In the case of glycerine, due to the lack of nitrogen, the C/N molar ratio was not calculated. The high nitrogen content suggests that potato wastewater can be applied as a nitrogen source as a substitute for peptone or yeast extract, which are expensive. In turn, due to the sugar content, apple pomace can be used as a carbon source for microorganisms, so does glycerine — a source of glycerol. Because of the low content of separate substrates in these residues — nirogen (pomace and glycerine) and carbon (potato wastewater), it could be assumed the the individual use of potato wastewater, apple pomace, or glycerine will not provide beneficial results. It would result in a very high or very low molar ratio of carbon to nitrogen in the growth environment, which in turn would limit the metabolic activity of microorganisms. It should be remembered that the metabolism of PAB is influenced by the growth conditions. The simultaneous effective production of metabolites and growth of PAB are ensured by C/N molar ratio in the range of 4:1–8:1 (Piwowarek et al. 2019). Therefore, 10 variants of media were used in this study to check which industrial residues and in what proportions have the most beneficial effect on the metabolism of the tested strain.

The consumption of macroelements contained in the culture medium, i.e., carbon or nitrogen, indicates their assimilation and/or fermentation by microorganisms. The *P. freudenreichii* DSM 20271 strain tested in this study consumed glucose, fructose, and/or glycerol present in the media, but in neither of the analyzed variants, 100% of carbon sources was consumed. These results are in line with the literature data, which have shown that PAB cultivated in media containing residues did not completely use the carbon sources from the by-products. In a study conducted by Feng et al. (2011), the *P. freudenreichii* CCTCC M207015 strain consumed 75–82.80% (depending on the cultivation variant) of carbon sources derived from sugar cane molasses (glucose, fructose, sucrose). In turn, the *Propionibacterium acidipropionici* ATCC 4875 strain utilized 92% of sugars (glucose, fructose) in the medium containing the hydrolysate of Jerusalem artichoke (Liang et al. 2012). Sarmiento-Vásquez et al. (2021) cultivated *Propionibacterium jensenii* DSM 20274 in a medium containing used cocoa pod husk hydrolysate and pure glycerol. During 156 h of fermentation, the tested strain consumed 89% of glucose and 39% of glycerol. In the study by Ammar et al. (2020), the *P. freudenreichii* DSM 4902 strain used for the fermentation of sweet sorghum bagasse (SSB) hydrolysate consumed 87% of glucose as a carbon source. The authors also cultured the DSM 4902 strain in media containing various doses of SSB hydrolysate and pure laboratory glycerol — in none of the variants, the total consumption of glucose (with SSB) or glycerol by the tested strain was observed. The differences in the use of carbon sources may result from the variations in their initial concentrations, culture conditions (temperature, pH, type of residue), or the strains used. The incomplete use of carbon sources by the DSM 20271 strain may be due to the fact that the cultures were carried out in media consisting only of industrial residues, without additional supplementation, which made it difficult for the bacteria to quickly adapt to growth conditions. It seems that extending the fermentation process by 24–48 h would allow increased utilization of sugars or glycerol by the tested strain. Microorganisms must adapt to the environment to exhibit efficient metabolism, which in turn depends on access to nutritional sources and the possibility of their use. The residues used in this study could contain substances that can limit the metabolism of PAB (e.g., pesticides, heavy metals, and polyphenols) (Kiczorowska et al. 2006; Góralczyk et al. 2009; Kaveli et al. 2015; Li et al. 2015, 2020). Apart from glycerol, waste glycerine contains methanol and free fatty acids (Pawlicka-Kaczorowska and Czaczyk 2017), which may limit the metabolism of microorganisms, including their uptake of nutrients from the growth environment.

Pyruvate is an important component in the metabolic pathways of PAB. It acts as a substrate for the production of propionic and acetic acid as well as biomass. It has been shown that during the fermentation or cofermentation of glucose or/and glycerol, bacteria prefer sugar to form biomass and glycerol for the production of propionic acid (Liu et al. 2011; Wang and Yang 2013; Ammar et al. 2020). Bacteria of the *Propionibacterium* genus produce pyruvate via two pathways: Embden-Meyerhof-Parnas (EMP) and hexose monophosphate (HMP). In both these pathways, glucose is used by the bacteria. During the fermentation of glucose, over 90% of pyruvate is produced by bacteria via the EMP pathway, and approximately 10% by the HMP pathway (Wang and Yang 2013). The HMP pathway involves the reduction of 11 mol of NAD^+^, resulting in the formation of 11 mol of NADH, 5 mol of pyruvate, and 5 molecules of ATP. This pathway is mainly used by bacteria for the synthesis of ATP, which is needed for the multiplication of bacterial cells (Wang and Yang 2013). Glycolysis (glucose as a carbon source) results in the formation of 2 mol of pyruvate and NADH and 2 ATP molecules. Glycerol cannot be used via HMP pathway. This carbon source is converted by PAB to pyruvate (1 mol) to form ATP and 2 mol of NADH. For the growth of biomass, PAB require 4 mol of pyruvate, 33.7 ATP, and 5.75 NADH, which can be provided faster by the HMP pathway (and even EMP) and glucose as a carbon source compared to glycerol (Wang and Yang 2013). This would explain the more intensive growth of *P. freudenreichii* DSM 20271 in the media in which sugars constitute at least half of the carbon sources.

Based on the results obtained in this study, it can be concluded that the *P. freudenreichii* DSM 20271 strain can grow in media containing the tested residues — as long as there is apple pomace and/or potato wastewater in the medium, waste glycerine does not constitute a favorable environment for the growth of PAB. Potato wastewater, which is a source of nitrogen, seems to be of the greatest importance for bacterial growth. The fact that the growth of PAB was abundant in the media containing potato wastewater proves that this material contained nitrogen sources that can be absorbed by PAB (amino acids, amines, peptides). Some amino acids, mainly arginine and aspartic acid, act as a buffer, stabilizing and enhancing the kinetics of fermentation by reducing the inhibitory effect of the acids produced by PAB (eliminating the negative feedback effects) (Guan et al. 2013). Fröhlich-Wyder et al. (2002) reported that the deficiency of aspartic acid in the medium inhibits the growth of PAB.

### Production of acids

The most favorable parameters for the production of propionic acid were achieved in M7 (8.15 g/L, yield: 0.48 g/g, productivity: 0.068 g/L/h), which contained potato wastewater, sugars (9.92 g/L, from apple pomace), and glycerol (9.21 g/L, from waste glycerine). The production yield of acetic acid in M7 was 0.13 g/g. The P/A ratio achieved in this medium was 3.68:1. During the synthesis of propionic acid, 2 mol of NADH are oxidized to 2 mol of NAD^+^, and during the synthesis of acetic acid, 1 mol of NADH is produced. Since glycolysis (glucose as a carbon source) cannot provide enough NADH for the bacteria to produce propionic acid, acetic acid is formed to enable an additional reduction step in order to maintain the intracellular redox balance. The production of propionic acid from glucose, which has a lower degree of reduction (4.00) than propionic acid (4.67), requires the co-production of a more oxidized (compensating) metabolite — acetic acid (4.00). Glycerol has the same degree of reduction (4.67) as propionic acid (4.67), and therefore, its conversion to pyruvate generates enough NADH for the biosynthesis of propionic acid, without the need for the co-production of acetic acid (Wang and Yang 2013), which is manifested by a higher yield of propionic acid and a more favorable P/A ratio. Theoretically, fermentation of sugars can result in a P/A ratio of 2:1. The use of glycerol as a carbon source increases this ratio up to 8.42:1 (Ammar et al. 2020). In M7, the P/A ratio was 3.68:1, which is higher than the theoretically ratio resulting from glucose and lower than that from glycerol. It is most likely that propionic acid was made by bacteria using two types of carbon sources: sugars and glycerol. Sugars were also used to form biomass. The highest biomass yield was achieved in M7, which in turn stimulated the production of acetic acid to maintain the intracellular redox balance. During the formation of biomass, approximately 5.75 NAD^+^ is produced (Wang and Yang 2013), resulting in increased synthesis of acetic acid (during which NAD^+^ is reduced to NADH, to maintain the intracellular redox balance). In all media containing mostly sugars as carbon sources, the P/A ratios were close to 2:1 (M1 — 2.26:1, M2 — 2.54:1, M4 — 2.42:1, M8 — 3.10:1, M9 — 2.51:1). In the remaining variants, in which glycerol was the major component (or in equal proportion as sugars), the P/A ratios were close to 4:1. These results confirm that glycerol, if present in the medium, was used for the production of propionic acid, while sugars for both — building biomass and synthesis of acids.

Ammar et al. (2020) used three different proportions of carbon sources in the media (SSB hydrolysate/glycerol: 2:1, 1:1, 1:2). As the amount of glycerol increased, both propionic acid yield (0.54, 0.57, and 0.59 g/g, respectively) and P/A ratio (4.72:1, 5.07:1, and 8.42:1, respectively) increased. In the present study, the ratio of sugars to glycerol in M7 was 1:1. With the same ratio of carbon sources, a lower yield (0.48 g/g) and a lower P/A ratio (3.68:1) were achieved in this study compared to the work of Ammar et al. (2020). This difference in yield and the P/A ratio could be due to the following reasons: (1) Ammar et al. (2020) used media containing a higher concentration of carbon sources (30 g/L), regardless of the variant; (2) the authors used supplements containing vitamins, microelements, and nitrogen (e.g., yeast extract and peptone); and (3) they used pure laboratory glycerol (in this study, waste glycerine was used). Due to the presence of impurities, glycerol derived from waste glycerine is characterized by a lower efficiency of propionic acid production. Kośmider et al. (2010) carried out propionic acid fermentation with *P. freudenreichii* using pure glycerol or waste glycerine. Regardless of the initial concentration of carbon source (20 and 40 g/L), more efficient fermentation of propionic acid was achieved in the medium containing laboratory glycerol (0.68 and 0.45 g/g, respectively). The yields achieved in the media with waste glycerine were 0.56 and 0.25 g/g, respectively.

One of the factors limiting the microbial production of propionic acid on an industrial scale is the by-products of PAB culture, mainly acetic acid. The extraction of propionic acid by distillation is strongly hindered by this carboxylic acid. A low concentration of acetic acid increases the efficiency of extracting pure propionic acid from the culture fluid (Barbirato et al. 1997; Ammar and Philippidis 2021). The results obtained in this study are quite promising in the context of limiting the synthesis of acetic acid. The findings indicate that the efficiency of acetic acid synthesis is decreased when a higher concentration of glycerol is used. In order to increase the efficiency of the extraction and purification of propionic acid, a higher concentration of glycerol could be used from the waste glycerine at the expense of apple pomace. However, fruit residues should not be completely abandoned. In M6 (glycerine + potato wastewater, glycerol-to-sugar ratio — 7:1), the highest P/A ratio (4.11:1) was achieved compared to other variants, but the efficiency of synthesis (0.42 g/g) was lower compared to M7 (0.42 g/g, P/A ratio: 3.68:1). This suggests that pomace should be used in combination with glycerine and potato wastewater. In M10, in which the glycerol-to-sugar ratio was approximately 2.65:1 (18.33–6.91 g/L), the efficiency of propionic acid production was 0.35 g/g and the P/A ratio was 3.73:1. The studies by Ammar et al. (2020) showed that an increase in the mass ratio of glycerol to sugars to 2:1 caused a significant increase in both the efficiency of propionic acid production and the P/A ratio — this effect was not observed in this study. This is probably due to the fact that a small amount of potato wastewater was used in M10, which resulted in a low nitrogen concentration in the environment and a high molar C/N ratio. As a consequence, the tested strain showed a limited growth potential and thus limited metabolism. Both propionic acid production and cell growth are related to metabolic pathways that include phosphorylation at the substrate level and maintain intracellular redox balance (glycolysis, pentose phosphate pathway, Wood-Werkman pathway). The results obtained in this study confirm the importance of selecting appropriate amounts of residues to ensure that microorganisms have access to individual nutrients at optimal concentrations.

**Fig. 4:**
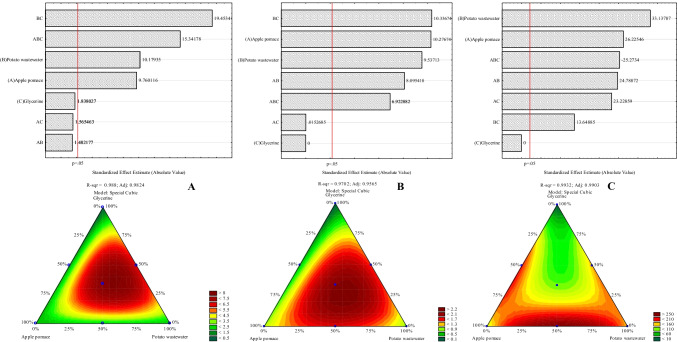
Pareto diagrams and contour plots — the influence of the investigated factors on the production of metabolites of *P. freudenreichii* DSM 20271 strain in 120 h of culture: propionic acid (**A**), acetic acid (**B**), vitamin B12 (**C**)

Considering individual residues, potato wastewater had the greatest impact on propionic acid production, followed by apple pomace (Fig. 4A). Waste glycerine, when used as the sole component of the medium, had no major influence on the synthesis of propionic acid. This is due to the fact that waste glycerine lacked vitamins or nitrogen sources, which prevents the growth of bacteria and inhibits other metabolic processes. Among the studied variants, the environment that allowed the most efficient synthesis of propionic acid was the medium containing several residues, including waste glycerine, as well as potato wastewater (mainly), and apple pomace. Potato wastewater and apple pomace are important for propionic acid production, even when used alone, because both these residues contain carbon and nitrogen sources (even when used in small amounts). Perhaps, these residues, or at least one of them, also contain stimulating substances that are essential for the production of propionic acid (e.g., biotin and microelements). The availability of biotin is particularly important for the synthesis of propionic acid by PAB (Falentin et al. 2010; Wang and Yang 2013). Vitamin B7 is involved in the Wood-Werkman cycle, a propionic acid synthesis pathway. Together with oxaloacetate carboxyltransferase, this vitamin takes part in the conversion of pyruvate to oxaloacetate and L-methylmalonyl-CoA to propionyl-CoA. Both apples and potatoes contain, for example (apples-potatoes): biotin (20 ng/100 g–1 µg/100 g) (Staggs et al. 2004), Fe (0.1–3 mg/100 g), Ca (3–30 mg/100 g), K (98–413 mg/100 g), Na (10 mg/100 g, only potatoes), Zn (0.02–0.35 mg/100 g), Mn (0.04–0.60 mg/100 g), and Mg (4.7–23 mg/100 g) (according to the U.S. Department of Agriculture, FoodData Central). The presence of these microelements in plant raw materials may be due to their presence in industrial residues — considering the amount of these compounds in the raw material.

Most of the industrial by-products used for the production of propionic acid under laboratory conditions require pretreatment (chemical or enzymatic hydrolysis) and/or supplementation with carbon sources (e.g., pure glycerol), nitrogen sources (e.g., yeast extract, tryptone, and peptone), minerals (e.g., ZnCl_2_, FeCl_2_, MnCl_2_, and CaCl_2_), or vitamins (e.g., riboflavin, pantothenic acid, biotin, thiamine, and cyanocobalamin) (Liang et al. 2012; Zhu et al. 2012; Kagliwal et al. 2013; Stowers et al. 2014; Wang et al. 2017; Ammar et al. 2020). Some previous studies have reported a higher efficiency of propionic acid production compared to the present study. Ammar et al. (2020) achieved an efficiency of 0.54–0.59 g/g (*P. freudenreichii*, SSB). Similar results were reported by Kagliwal et al. (2013) (*Acidipropionibacterium acidipropionici*, former *Propionibacterium acidipropionici*, wheat flour) and Stowers et al. (2014) (*A. acidipropionici*, corn mash). On the other hand, some studies have reported a lower or comparable yield of propionic acid synthesis. For example, depending on the medium used, Liang et al. (2012) achieved an efficiency of 0.38–0.48 g/g (*A. acidipropionici*, Jerusalem artichoke hydrolysate). Similar values were also reported by Zhu et al. (2012) (0.29 and 0.37 g/g, *A. acidipropionici*, sugarcane bagasse hydrolysate) and Wang et al. (2017) (0.44–0.50 g/g, *A. acidipropionici*, corn stover hydrolysate).

In this study, depending on the medium used, the yield of propionic acid varied from 0.14 to 0.48 g/g. In four of the analyzed media variants (M2, M4, M6, M7), the production yield exceeded 0.40 g/g. The use of three different residues carries the risk of contaminating the growth environment with substances that may limit the metabolic properties of bacteria (fatty acids, methanol, heavy metals, polyphenols). It should also be noted that supplements such as yeast extract, peptone, or vitamin preparations are composed in accordance with the requirements of microorganisms, with all the necessary compounds in optimal doses, which cannot be provided by residues. Thus, it seems that the media used in this study, especially M7, is promising for the industrial production of propionic acid.

Industrial production of propionic acid involves the use of chemical processes that are more cost-effective than microbial synthesis. Therefore, there is a need to develop methods that will increase the profitability of biotechnological processes. One such approach is the utilization of industrial residues, which are cheap and readily available raw materials. Considering that the medium is one of the major factors affecting the cost of propionic acid production (Yang et al. 2018), the use of residues as components of culture media seems to be an excellent solution. The above-presented literature data (Liang et al. 2012; Zhu et al. 2012; Kagliwal et al. 2013; Stowers et al. 2014; Wang et al. 2017; Ammar et al. 2020) indicate that residues require enzymatic treatment and are usually used as a carbon source, and hence they should be supplemented often with expensive laboratory preparations such as nitrogen sources and vitamins (Yang et al. 2018; Piwowarek et al. 2021b). Importantly, in this study, the DSM 20271 strain was cultivated in the media containing only industrial residues, without any added supplements. The residues were used without any pretreatment. Therefore, the obtained efficiency (0.48 g/g), with low costs of the medium, suggests that the use of residues may be an alternative to the chemical production of propionic acid (Piwowarek et al. 2021b).

In addition to the economic benefits, the proposed production method of propionic acid would also be beneficial from an environmental point of view. In recent years, politicians, ecologists, and researchers have been showing an increased interest in sustainable development and zero-waste approaches in order to keep industrial residues in the economic cycle for as long as possible (Singh et al. 2017). Pomace from fruit processing, residues resulting from potato processing, and waste glycerine are considered as materials affecting the environment. However, due to their rich and varied composition, these by-products may be valuable components of microbiological media. Fermentation allows the use of industrial by-products as components of microbiological media, enhancing the synthesis of propionic acid and other metabolites of PAB (e.g., cobalamin) which can be used in various industries. Moreover, these valuable metabolites can be synthesized using *P. freudenreichii* bacteria, which are microorganisms widely applied in food production or animal nutrition. In summary, it seems that fermentation may be a cheap, energy-saving, and sustainable alternative for managing of industrial side-streams.

### Production of vitamin B12

The obtained results and statistical analysis of DoE (Fig. 4C) revealed that potato wastewater was the most important side-stream for cobalamin production by the studied strain, followed by apple pomace. The presence of glycerine in the medium, at the expense of the other residues used in this study, resulted in lower content of vitamin B12 in the bacterial biomass. Kośmider et al. (2012) concluded that waste glycerine serves as an excellent carbon source for the synthesis of vitamin B12 by *P. freudenreichii* 1 strain. These authors used the growth media enriched with various amounts of supplements, including biotin, Ca pantothenate, CoSO_4_·6H_2_O, NaH_2_PO_4_·2H_2_O, DMBI, and casein hydrolysate, depending on the variant. The biosynthesis of vitamin B12 by *P. freudenreichii* 1 is influenced by the following compounds, depending on their concentrations: Ca pantothenate, NaH_2_PO_4_·2H2O, casein hydrolysate, and glycerol. In the study by Kośmider et al. (2012), glycerine seemed to have served as a carbon source for bacterial growth or propionic acid production. Supplements, on the other hand, provided bacteria with the compounds essential for the production of cobalamin. In the present study, potato wastewater and apple pomace acted as supplements promoting the synthesis of vitamin B12, as they likely provide vitamins and microelements needed for PAB metabolism.

Potatoes contain riboflavin (0.038 mg/100 g), nicotinamide (1.03 mg/100 g), thiamine (0.021 mg/100 g), pantothenic acid (0.302 mg/100 g), pyridoxine (0.239 mg/100 g), and folic acid (17 µg/100 g) (according to the U.S. Department of Agriculture, FoodData Central). Fresh apples contain 0.008–0.2 mg/kg cobalt (Kabata-Pendias and Pendias 1999; Langauer-Lewowicka and Pawlas 2012). Apples (regardless of the variety) are also a reservoir of vitamins, including group B (riboflavin — 0.074 mg/100 g, nicotinamide — 0.074 mg/100 g, thiamine — 0.009 mg/100 g, pyridoxine — 0.033 mg/100 g, folic acid — ≤ 6 µg/100 g) (according to the U.S. Department of Agriculture, FoodData Central). Riboflavin is a precursor of DMBI, which is necessary for the synthesis of active vitamin B12. Cobalamin production by the tested strain in the analyzed media suggests that the precursors of this compound (cobalt, riboflavin), which are derived from fresh raw materials, are present in the residues used in this study.

Chamlagain et al. (2016) cultivated various strains of PAB for the synthesis of vitamin B12. One of the media, which also served as the basis for other variants, was the WBM model medium. The other variants were added with the precursors of vitamin B12 — DMBI or riboflavin and/or nicotinamide (nicotinamide and riboflavin are the precursors of dimethylbenzimidazole — DMBI) (Deptula et al. 2015). Depending on the strain, the production of cobalamin in nonsupplemented media ranged from 25 to 204 µg/g wet biomass. The highest content of vitamin B12 in bacterial biomass was determined in the variant added with DMBI (over 250 µg/g wet biomass). In this study, based on the medium variant, the DSM 20271 strain produced 0.9–2.9 µg/g vitamin B12, which significantly differs from the value reported by Chamlagain et al. (2016). This is due to the fact that the medium used by these authors (WBM) is specifically designed for PAB, providing them with all the necessary ingredients (whey extract, yeast extract, sodium D/L-lactate syrup, Tween 80, magnesium sulphate, manganese (II) sulphate, potassium phosphate buffer) in optimal concentrations. Furthermore, the addition of precursors promoting vitamin B12 synthesis also intensified the synthesis of this metabolite. Although the environment favors the production of cobalamin by the tested strain, the by-products of the technological processes used do not allow achieving vitamin B12 at a level similar to clean laboratory media. This is probably due to the insufficient amount of individual precursors of this metabolite in the used raw materials. The difference in the final concentration of vitamin B12 in the bacterial cell biomass may also be attributed to the variations in the applied culture conditions. Chamlagain et al. (2016) conducted cultures for 168 h (up to 72 h — anaerobic conditions, 72–166 h — aerobic conditions), which is favorable for the synthesis of cobalamin by PAB. In this study, fermentation lasted 120 h — extension of the culture and aeration of the growth environment may increase the efficiency of vitamin B12 production in the given industrial residues.

Almost the entire industrial demand for vitamin B12 is met by microbiological production, mainly with the use of genetically modified bacterial strains of *Pseudomonas denitrificans* species. The use of genetically modified organisms for the production of food and other common products is still under debate, especially in public perception. There is an ongoing search for other, cost-effective ways to obtain this compound using microorganisms, but without modifying their genome. Industrial strain of *P. denitrificans* is able to produce vitamin B12 at the level of 214 mg/L (Li et al. 2008), it is much more compared to production of this compound in this study by *P. freudenreichii* DSM 20281 strain from side-streams. The advantage of the tested strain is that *Propionibacterium freudenreichii* is the only microorganism with GRAS status (Thierry et al. 2011) that can synthesize the active form of vitamin B12, and is therefore considered as a unique candidate for the microbiological enrichment of food products (e.g., veg products) and feed with this vitamin. The profitability of this process can be increased by using post-production residues as a matrix. The obtained results show that *P. freudenreichii* can produce vitamin B12 in media containing industrial residues only, including edible, plant origin side-streams (fruit pomace, potato wastewater). This creates the prospect of creating a vegan food product using plant residues, which would contribute to zero-waste policy and sustainable development. For this purpose, we have to apply side-streams that would guarantee the efficient production of vitamin B12 by PAB and which would be safe and acceptable to consumers, e.g., fruit residues — apple, grape, and berry pomace. Moreover, the tested strain can produce vitamin B12 from apple pomace and potato wastewater without any supplementation (it is important because the addition of cobalt and/or DMBI to growth environment is not allowed in the case of food production) (Chamlagain et al. 2018). This suggests that the given residues provide the bacteria with the necessary nutrients. On the other hand, it should be remembered that riboflavin can be synthesized by PAB if it is not available in a growth environement (Falentin et al. 2010; Piwowarek et al. 2021c). Therefore, the residues should be checked for the content of individual compounds important in the context of vitamin B12 production — to ascertain their origin.

This research focused on the utilization of industrial residues (apple pomace, waste glycerine, potato wastewater) for the production of propionic acid and vitamin B12 by *P. freudenreichii* DSM 20271 strain. The obtained results showed that the above residues, when used in appropriate proportions, promote the growth of *P. freudenreichii* bacteria. The fact that the tested strain produced both propionic acid and vitamin B12 proves that the used residues contain nutrients that are available for bacteria, such as carbon sources (sugars, glycerol), nitrogen sources, vitamins, and microelements. The industrial side-streams used did not require any pretreatment or additional supplementation (often with expensive laboratory preparations), and the achieved efficiency of propionic acid or vitamin B12 synthesis was comparable to or even higher than other documented results (with hydrolysis and enrichment of media with clean laboratory preparations). It seems that microbiological management of the analyzed by-products using PAB is an ideal alternative to obtain metabolites of these microorganisms. Moreover, biotechnological disposal of residues will contribute to protecting the environment. Despite the promising results observed in this study, it is necessary to improve the fermentation of residues, for example, by immobilizing microorganisms, allowing the bacteria to adapt to the culture environment to counteract the negative feedback of the produced acids, or using bioreactor culture.

## Data Availability

Data available on request.
